# Circulating Fatty Acids Associate with Metabolic Changes in Adolescents Living with Obesity

**DOI:** 10.3390/biomedicines12040883

**Published:** 2024-04-17

**Authors:** Branko Subošić, Jelena Kotur-Stevuljević, Nataša Bogavac-Stanojević, Vera Zdravković, Maja Ješić, Smiljka Kovačević, Ivana Đuričić

**Affiliations:** 1 Biochemical Laboratory, University Children’s Hospital, 11000 Belgrade, Serbia; branko.subosic@udk.bg.ac.rs; 2 Department of Medical Biochemistry, Faculty of Pharmacy, University of Belgrade, 11221 Belgrade, Serbia; jelena.kotur@pharmacy.bg.ac.rs (J.K.-S.); natasa.bogavac@pharmacy.bg.ac.rs (N.B.-S.); 3 Department of Endocrinology, Medical Faculty, University Children’s Hospital, 11000 Belgrade, Serbia; vera.zdravkovic@med.bg.ac.rs (V.Z.); maja.jesic@med.bg.ac.rs (M.J.); smiljka89kovacevic@gmail.com (S.K.); 4 Department of Bromatology, Faculty of Pharmacy, University of Belgrade, 11221 Belgrade, Serbia

**Keywords:** childhood obesity, fatty acids, telomere length, lipids, redox status

## Abstract

Fatty acids play a crucial role in obesity development and in the comorbidities of obesity in both adults and children. This study aimed to assess the impact of circulating fatty acids on biomarkers of metabolic health of adolescents living with obesity. Parameters such as blood lipids, redox status, and leukocyte telomere length (rLTL) were measured alongside the proportions of individual fatty acids. The Mann–Whitney U test revealed that individuals with obesity exhibited an unfavorable lipid and redox status compared to the control normal weight group. The group with obesity also had lower plasma n-3 polyunsaturated fatty acids (PUFAs) and a higher ratio of n-6 to n-3 PUFAs than the control group. They also had a shorter rLTL, indicating accelerated biological aging. There was an inverse association of rLTL and plasma n-6-to-n-3 PUFA ratio. Future studies should explore the impact of recommended nutrition plans and increased physical activity on these parameters to determine if these interventions can enhance the health and well-being of adolescents with obesity, knowing that early obesity can track into adulthood.

## 1. Introduction

Fatty acids (FAs) are naturally present in foods that contain fats, mainly in the form of triglycerides. The composition of FAs in the diet can vary depending on the sources of dietary fat, food processing methods, and cooking techniques. Obesity is most often associated with an increased intake of calorie-dense foods (rich in fats and refined carbohydrates) and a lack of physical activity. According to the World Obesity Federation, globally, 175 million children and adolescents aged 5–19 years were living with obesity in 2020, with a forecast that this number will reach 383 million by 2035 [1]. FAs are molecules that serve as building blocks of complex fats (e.g., triglycerides and phospholipids) and play a crucial role in energy storage and metabolism [2]. In obesity, FAs contribute to metabolic complications through various mechanisms. One is adipose tissue dysfunction linked with insulin resistance (IR) [3]. Adipose tissue stores excess energy in the form of triglycerides, and the hypertrophy of adipose tissue manifests significant alterations in cell metabolism. Basal lipolysis is elevated in hypertrophic adipocytes [4], thus increasing the release of non-esterified fatty acids (NEFAs) into the bloodstream. Furthermore, hypertrophic adipocytes show IR, leading to impaired insulin-dependent glucose uptake and higher blood glucose levels [5]. In response, the pancreas produces more insulin, exacerbating fat storage and promoting weight gain [6]. In addition to IR, due to increased lipolysis, fatty acids and cholesterol are accumulated in ectopic sites that are not the primary places of fat deposition, and lipotoxicity occurs [7]. A chronic state of low-grade inflammation accompanies obesity. This inflammation is associated with a number of pro-inflammatory molecules, including chemokines and cytokines, which can disrupt normal metabolic processes, induce IR, and contribute to the differentiation of preadipocytes into mature adipocytes [8]. Inflammation can also influence appetite regulation, mainly through the Toll-like receptor 4, potentially leading to overeating [9]. High levels of NEFAs can disrupt mitochondrial function within cells. Mitochondria are the key cellular site of fatty acid oxidation [10] and, hence, they are vital for the removal of fatty acids, partitioning them away from ectopic accumulation as triglycerides. In a state of negative energy balance, white adipose tissues release NEFAs for fatty acid oxidation in the liver and muscles, providing energy. In contrast, excess energy intake leads to adipocyte hypertrophy and hyperplasia, increased NEFA release, mitochondrial stress, IR development, and ectopic fat deposition [11]. Primary mitochondrial disorders also affect fat storage, causing symmetrical lipomatosis [12]. Excessive NEFAs accumulate in the liver, leading to non-alcoholic fatty liver disease (NAFLD). NAFLD can further exacerbate IR and metabolic dysfunction, contributing to overall obesity-related health issues. Untreated obesity creates a cycle where pathophysiology leads to more weight gain and additional fat storage [13]. This positive feedback loop can make it increasingly difficult for individuals, including children and adolescents, to lose weight and maintain a healthy body composition. Notably, although NEFAs are a normal part of lipid metabolism per se, not all contribute to the comorbidities of obesity in the same way.

Based on saturation level, fatty acids are considered as saturated (SFAs), monounsaturated (MUFAs), and polyunsaturated fatty acids (PUFAs) [14]. SFAs have no double bonds between the carbon atoms in their chain. They are usually solid at room temperature and are mainly found in animal fats and tropical oils. The most common SFAs in the human diet are palmitic (16:0) and stearic acid (18:0), with palmitic acid being more atherogenic. MUFAs have one double bond in their hydrocarbon chain. They are typically liquid at room temperature and are found in olive oil, avocados, and nuts. MUFAs are considered healthy fats [15]. PUFAs have multiple double bonds in their chain. They are liquid at room temperature and include two main types: omega-3 (n-3) and omega-6 (n-6) FAs. Essential linoleic acid (LA, 18:2n-6) and α-linolenic acid (ALA, 18:3n-3) are found in high proportions in foods of plant origin (plant (seed) oils, some seeds, and nuts). The metabolic products of LA and ALA metabolism are arachidonic acid (AA, 20:4n-6), eicosapentaenoic acid (EPA, 20:5n-3), and docosahexaenoic acid (DHA, 22:6n-3), respectively [16]. The most important direct source of EPA and DHA is oily fish (e.g., sardines, mackerel, salmon). A higher intake of n-6 relative to n-3 PUFAs, which is typical for the Western diet, can contribute to inflammation, oxidative stress, and tissue damage [17]. Likewise, a high intake of SFAs or an inadequate ratio of saturated to unsaturated fats can promote cellular stress and metabolic dysregulation. The balance between total n-6 PUFAs and total n-3 PUFAs is crucial. Previous research suggests that the optimal ratio of total n-6 to total n-3 PUFAs is less than 5:1, or even lower than this, in order to maximize the benefits of individual fatty acids [18]. Mild inflammation and the creation of excess reactive oxygen species (i.e., oxidative stress) hasten cellular aging, evident in the reduction of telomere length, an inherent characteristic in obesity [19]. Telomeres, characterized by hexanucleotide sequences (TTAGGG) at the termini of chromosomes, diminish with every cellular division, initiating malfunction in the cell cycle upon reaching a crucial threshold of shortening [20]. This results in potential genomic instability and cellular demise. Obesity, distinguished by widespread inflammation and the generation of oxidative stress, significantly influences DNA integrity and telomere length. Even in pediatric instances, parameters related to adiposity demonstrate an inverse correlation with telomere length [21].

Certain genetic predispositions, unhealthy dietary habits, and low levels of physical activity play an important role in the development of childhood and adolescent obesity. The main goal of this study was to assess the circulating levels of fatty acids and their associations with specific biochemical, redox, and genetic parameters in adolescents living with obesity. Understanding the interplay between fatty acids and various physiological markers can offer critical information regarding the underlying mechanisms contributing to obesity onset and development among adolescents.

## 2. Materials and Methods

This research was designed as an observational case-control study with 91 obese and 44 healthy non-obese adolescents. To ensure comparability between controls and patients and to avoid the main confounders, the adolescents were matched by age and gender. Our study followed the age and gender characteristics of obese adolescents in the Republic of Serbia according to the Serbian National Health Survey 2019 [22]. Under these conditions, our sample meets the criteria of representativeness, i.e., the sample reflects the demographic and other relevant characteristics of the target population. The sample size was obtained by rejecting the null hypothesis using a 2-tailed test, with α = 0.05, β = 0.2, medium effect size of 0.5, and allocation ratio of N2/N1 0.5. The sample size was further increased by 15% in each group according to skewed variable distribution [23]. G*Power software version 3.1.9.4 (Universität Kiel, Kiel, Germany) was applied to determine the number of study subjects. Data and samples from all participants were collected at the University Children’s Hospital. Approval was obtained from the Institutional Ethical Board of the University Children’s Hospital (Ethical License No. 16–25, 10 June 2022). All patients included in this study were adolescents having their first medical examination by a pediatric endocrinologist due to obesity. Patients meeting the inclusion criteria (obesity as the only diagnosis using age (10–18 years) and sex-specific body mass index (BMI), without any other comorbidities) were selected and included in this study after providing written consent. The controls included healthy adolescents having regular annual medical examinations, and these individuals had a BMI < the 95th percentile, defined for age and sex, and no acute or chronic metabolic conditions. Fasting blood samples were collected as part of routine medical checkups for patients. Serum, plasma, and peripheral blood mononuclear cells (PBMCs) were isolated from blood by centrifugation.

### 2.1. Measurement of Blood Lipids and Associated Parameters

Most of the biochemical parameters were determined from serum isolated from blood by centrifugation at 3500 rpm in a Rotofix 32 A type of centrifuge (Andreas Hettich GmbH & Co. KG, Tuttlingen, Germany). Lipid parameters (total cholesterol, high-density lipoprotein (HDL)-cholesterol and triglycerides (TGs)) were measured using standard enzymatic methods, while low-density lipoprotein (LDL)-cholesterol was determined using the Friedwald equation (total cholesterol—HDL-cholesterol—TGs—2.2). Biochemical analyses were performed on Siemens EXL 200 and Siemens RxL MAX analyzers (Siemens Healthcare Diagnostics Inc., Newark, DE, USA). Insulin was measured by a chemiluminescent magnetic microparticle immunoassay on an Architect i1000 sr analyzer (Architect: Abbott Laboratories, Irving, TX, USA). The obtained results were used to calculate two parameters: risk for cardiovascular diseases (RFCVD; total cholesterol—HDL-cholesterol) and index of atherosclerosis (IA; LDL-cholesterol—HDL-cholesterol).

### 2.2. Assessment of Redox Status Parameters

The values of antioxidant and prooxidative parameters were determined. Antioxidant factors included plasma or serum (where appropriate), total antioxidant status (TAS), superoxide dismutase (SOD) activity, and the concentration of total sulfhydryl groups (SHGs) as protective parameters. The prooxidative parameters were total oxidative status (TOS), advanced oxidation protein products (AOPPs), ischemia-modified albumin (IMA), and prooxidant–antioxidant balance (PAB). The methodologies used for the determination of oxidative stress parameters have been described previously. TAS was measured using Erel’s colorimetric method with ABTS as a chromogen [24]. SOD activity was measured according to the method of Misra and Fridovich [25], while SHG concentrations were determined by Ellman’s spectrophotometric method with the reaction of 2,2′-dinitro-5,5′-dithio-benzoic acid (DTNB) with aliphatic thiols [26] modified in our laboratory. An automated colorimetric method with ferrous ions was used for TOS quantification [27]. AOPPs were determined according to the Witko-Sarsat method using a reaction with glacial acetic acid and potassium iodide [28]. A semiquantitative spectrophotometric method, based on albumin and cobalt chloride, was used for IMA determination [29]. PAB was determined by a modified test using 3, 30, 5, and 50-tetramethylbenzidine as chromogen [30].

### 2.3. Analysis of Total Plasma Fatty Acid Composition

Plasma was used for the analysis of FAs. Direct in situ extraction and acid-catalyzed trans-esterification with 1.5 mL of 3 M HCl in methanol were applied to obtain fatty acid methyl esters (FAMEs). The mixture was vortexed and heated in a water bath at 85 °C for 45 min, then cooled, and hexane (Sigma Aldrich, Saint Louis, MO, USA) was added for the extraction of FAMEs [31]. Following centrifugation for 10 min at 3000 rpm, the upper hexane layer containing the FAMEs was transferred into vials and immediately analyzed. FAMEs were separated using gas chromatography on an Agilent 7890 instrument with flame ionozation detection. FAMEs were separated on a capillary column (CP-Sil88; 100 m × 0.25 mm, 0.2 μm film thickness; SUPELCO, Bellefonte, PA, USA) under the following conditions: 1 μL injection of the FAME mixture was made in split mode 20:1; the injector temperature was 250 °C with the injector split flow of 20 mL/min, the pressure of 31,623 psi, and the total flow of 24 mL/min; the oven temperature program started at 80 °C and increased by 4 °C/min up to 220 °C (hold time 5 min), then by 4 °C/min up to 240 °C, and then held at 240 °C for 10 min; the carrier gas (He) flow rate was adjusted to 1.0 mL/min and the makeup gas-nitrogen flow was adjusted to 25 mL/min; the FID detector temperature was 270 °C; and the run time was 55 min. Chromatographic peaks were identified by comparing their retention times with a standard FAME mix (Supelco FAME Mix, Bellefonte, PA, USA). The quantification was based on the ratio between each individual peak area and the sum of all peak areas. The results were expressed as the percentage of each individual fatty acid.

### 2.4. Measurement of Relative Leukocyte Telomere Length (rLTL)

PBMCs were isolated from EDTA blood by centrifugation using a Ficcol-Paque gel separator (GE Healthcare, Chicago, IL, USA). After washing with PBS, PBMCs were stored at −80 °C. DNA was isolated using a FlexiGene DNA kit, isopropanol precipitation, ethanol rinsing, and air drying. Isolated DNA was dissolved in FG3 buffer (hydration buffer). LTL was determined using real-time PCR with SYBR Green, incorporating two primers and measuring fluorescence. The method’s reliability was enhanced by using the albumin gene as a control, and the results are presented as a ratio of target gene cDNA to albumin gene cDNA [32].

### 2.5. Statistical Analysis

Data distribution was assessed through Kolmogorov–Smirnov or Shapiro–Wilk tests, as appropriate. Given the non-normal distribution of most parameters, data are presented as medians with 25th to 75th percentile values. The Mann–Whitney U test was used to test differences between independent samples. Spearman’s test was performed to evaluate correlations. The statistical significance threshold for all analyses was set at *p* < 0.05. The software package SPSS for Windows 18.0 (SPSS, INC., Chicago, IL, USA) was used for statistical analysis.

## 3. Results

The phenotypic characteristics of participants are presented in Table 1.

According to the data shown in Table 1, adolescents living with obesity had altered lipid parameters and calculated indexes compared to the control group (higher total and LDL-cholesterol, TGs, RFCVD, and IA, and lower HDL-cholesterol). While boys with obesity showed significant differences in these parameters compared to the control boys, the girls showed similar trends, albeit without statistical significance (Supplementary Table S1A,B). Except TAS, redox status parameters showed significant homeostasis disbalance with higher concentrations of prooxidant parameters (TOS, AOPPs, PAB) and a lower concentration of protective parameters (SOD, SHG) in the obese compared to the control group (including boys and girls). Remarkably, rLTL was shorter in those with obesity. The circulating fatty acids did not clearly indicate an imbalance of atherogenic and non-atherogenic FAs between the groups, but the values of EPA, docosapentaenoic acid, DHA, and total n-3 PUFAs were lower and the n-6-to-n-3 PUFA ratio was higher in those with obesity.

Spearman’s tests were performed to evaluate any significant correlations between the FAs and other parameters. The significant results are presented in Table 2.

There were positive correlations between the proatherogenic FA myristic acid (14:0) and blood TGs and algorithms for future cardiovascular risk in the obese group. Myristic acid also showed a positive correlation with the prooxidative redox status parameters TOS and AOPPs. The other proatherogenic FA, palmitic acid (16:0), showed a positive correlation with total and LDL-cholesterol. Linoleic acid (18:2n-6) also showed a positive correlation with TOS and AOPPs. There was no correlation of FAs with rLTL.

Table 3 shows the parameters that differed significantly between two groups of obese adolescents: one with an n-6-to-n-3 PUFA ratio greater than 5:1 (high) and the other with a ratio lower than this (low). The cut-off value of 5:1 aligns with the recommendations of different international organizations [18].

There was a significant difference in rLTL, a marker of biological aging, between groups with favorable and unfavorable n-6-to-n-3 PUFA ratios; rLTL was longer in the group with a lower ratio of n-6 to n-3 PUFAs. This group also had a higher proportion of non-atherogenic stearic acid (18:0), EPA (20:5n-3), and total n-3 PUFAs. In addition, the relationship between FAs and rLTL in the group of adolescents with a high n-6/n-3 PUFA ratio was analyzed using Spearman tests. The EPA showed a positive correlation (ρ = 0.267, *p* = 0.032), while 18:2n-6 showed a negative correlation (ρ = −0.319, *p* = 0.01) with rLTL.

## 4. Discussion

This study demonstrates that, even in adolescents, obesity is a condition characterized by significantly altered blood lipids and redox status markers. The blood lipids may be affected by the consumption of high-fat meals; increased endogenous synthesis (e.g., from excess dietary carbohydrate); decreased clearance from the bloodstream, for example, due to IR; and restricted metabolism due to a lack of physical activity [33]. Elevated levels of total cholesterol, LDL-cholesterol, and TGs, as well as decreased concentrations of HDL-cholesterol, were found in a group of adolescents living with obesity, which aligns with previous reports [34,35]. The results indicate that while a specific subset of boys with obesity shows significant differences in lipid parameters compared to non-obese boys, girls with obesity do not display statistically significant distinctions in lipid parameter concentrations compared to non-obese girls. The primary reason for these differences lies in the onset of sex hormone production during puberty. Boys experience an increased production of pro-atherogenic testosterone, resulting in elevated LDL-cholesterol and TG levels alongside reduced HDL-cholesterol. In contrast, girls undergo hormonal changes involving estrogen, which influences lipid parameter balance and aids in maintaining typical lipid levels despite obesity [36]. The proatherogenic lipid parameter of TGs positively correlated with myristic acid, while the proatherogenic lipid parameters of total and LDL-cholesterol positively correlated with palmitic acid. These findings suggest a metabolic link between these two SFAs and the more complex lipids. Myristic acid can increase the recruitment of newly synthesized triglycerides to lipoproteins such as VLDL and additionally reduce apoB-100 degradation, thereby increasing circulating TG concentrations [37]. Palmitic acid can suppress the expression of LDL receptors, reducing the clearance of LDL from the bloodstream, resulting in increased LDL-cholesterol concentrations [38]. The positive correlations of myristic acid with TGs and of palmitic acid with total cholesterol and LDL-cholesterol (Table 2) support these proposed links.

In addition to pro-atherogenic lipids, the protective component HDL-cholesterol may be affected by specific unsaturated fatty acids. One of the most damaging fatty acids is erucic acid (C22:1n-9), with a cardiotoxic effect primarily manifested as myocardial lipidosis (accumulation of lipids in the heart) [39,40]. Our results showed a strong negative correlation between HDL-cholesterol and erucic acid. It has been confirmed that HDL-cholesterol promotes direct cardioprotective effects mediated by interactions with the myocardium, particularly cardiomyocytes [41]. It enhances cardiac function by protecting against ischemia-reperfusion injury, reducing infarct size, and salvaging myocardial tissue. It improves cardiac function by minimizing infarct expansion and mitigating ventricular remodeling post-myocardial infarction [37]. Adolescents living with obesity had higher values of erucic acid compared to the control group, along with lower levels of HDL-cholesterol; these two observations may be linked [42]. Since there is altered oxidation present in conditions such as obesity, according to concentrations of AOPPs and TOS, the protective role of HDL-cholesterol has been compromised and these particles continue to degrade in the liver through the canonical ecto-F1-ATPase/PY213 pathway [43], leading to a decrease in the HDL-cholesterol level. Our results also suggest that circulating fatty acids, especially myristic acid, may contribute to CVD development, according to the values of RFCVD and IA, as indexes that include specific lipid parameters.

The potential roles of n-3 and n-6 PUFAs in cardiometabolic diseases have been recently reviewed [44]. TG levels, blood pressure, fasting blood glucose, HDL-cholesterol, and IR are all improved by n-3 PUFAs, probably explaining their protective effects on metabolic syndrome and CVD. Similarly, n-6 PUFAs, instead of SFAs, can positively impact blood lipids, especially LDL-cholesterol and IR [43]. On the other hand, an excessive intake of n-6 PUFAs with a low intake of n-3 PUFAs can predict obesity development [45]. Our results showed that adolescents living with obesity had lower n-3 PUFA levels and a higher n-6-to-n-3 PUFA ratio compared to the control group. This implies that heightened exposure to n-6 PUFAs during crucial developmental stages of adipocytes may lead to a lasting increase in adipocyte numbers. Consequently, this could increase the tendency to accumulate body fat [46].

In a study performed by DiNicolantonio and O’Keefe, a favorable n-6-to-n-3 ratio was set to be 4:1, or less, and it is believed that such a ratio is beneficial in preventing systemic inflammation and the development of metabolic syndrome [47]. Our results (Table 3) indicate that adolescents living with obesity with an unfavorable n-6-to-n-3 ratio have a shorter rLTL compared to those with a more optimal n-6-to-n-3 PUFA ratio. This observation strongly indicates that the low intake of n-3 relative to n-6 PUFAs causes cellular effects leading to accelerated cell aging. N-3 PUFAs regulate gene expression by modulating transcription factors like sterol-regulatory-element-binding proteins (SREBPs) and peroxisome proliferator-activated receptors (PPARs). Additionally, they influence epigenetic modifications, including histone alterations, DNA methylation, and miRNA levels, linked to gene repression or activation [48]. The current findings support the report of Liu et al. that a lower n-3 PUFA associates with shorter telomere length in children aged 3 to 4 years living with obesity [49]. Our observation extends this finding to those of older age. Furthermore, a more favorable n-6-to-n-3 PUFA ratio was linked to a higher proportion of the non-atherogenic stearic acid, EPA (20:5n-3) and a lower proportion of linoleic acid (18:2n-6). The results of the Spearman’s tests indicate significant correlations between certain fatty acids and rLTL in the context of high n-6/n-3 PUFA ratios. Higher levels of EPA and lower levels of linoleic acid are associated with longer telomeres when the ratio of n-6 to n-3 PUFAs is high (above 5:1). These novel findings suggest that specific fatty acids, such as EPA and linoleic acid, may have favorable effects on telomere length depending on the imbalance between n-6 and n-3 PUFAs. Linoleic acid has a beneficial role with moderate intake and when the n-6-to-n-3 PUFA ratio is low. In high-ratio states, linoleic acid loses its protective properties and is believed to contribute to a pro-inflammatory, pro-allergic, pro-thrombotic, and autoimmune-prone state [47]. A recent study reported that a higher dietary intake of DHA (22:6n–3) was significantly positively associated with rLTL in middle- to older-aged US males [50]. However, no significant associations were found between the intakes of total n–3 or total n–6 and rLTL. This research is based on dietary amounts of n–3 and n–6 PUFAs and not on their concentrations in blood, which is considered a more relevant marker for investigating the association with rLTL.

Oxidative stress is implicated in obesity and metabolic syndrome development, affecting insulin secretion and glucose transport in adipose tissue and muscles [51]. Locally generated reactive oxygen species damage cell structures, including lipids, proteins, and DNA. Excessive FA accumulation and cytokines induce systemic oxidative stress. Additionally, emerging evidence indicates a reduced systemic antioxidant defense system in patients with metabolic syndrome and obesity [52]. The current study (Table 1) shows higher prooxidant levels (TOS and AOPPs) in adolescents living with obesity than in the control group. Additionally, antioxidative protection is better in the control group of adolescents, indicated by higher SOD activity and SHG values compared to the obese group. An unexpectedly higher TAS was seen in the obese group; this could be explained by hyperuricemia in obesity. Hyperinsulinemia or IR may cause an impairment of the glycolytic pathway, leading to the accumulation of ribose-5-phosphate, a primary substrate for uric acid production. The observed positive correlations of linoleic acid and total n-6 PUFAs with TOS and AOPPs (Table 2) indicate that these FAs have an important role in oxidative stress development through the processes of non-enzymatic and enzymatic lipid peroxidation [53].

The fact that the analyses were performed in a relatively small study sample size should be acknowledged as a limitation of this study. Recruiting healthy adolescents with parental approval proved challenging, while the significant difference in circulating fatty acids between obese and normal-weight adolescents justified a smaller sample size. However, further research with a larger sample size is necessary to broaden our observations.

## 5. Conclusions

The results presented in this study illuminate obesity as a state marked by disrupted homeostasis in blood lipids and redox status, accompanied by a noteworthy reduction in telomere length. Elevated levels of proatherogenic lipids and decreased concentrations of protective HDL-cholesterol were observed in those with obesity, indicating a heightened risk of cardiovascular disease. Interestingly, high n-6-to-n-3 PUFA ratios were associated with accelerated cellular aging, with EPA and linoleic acid notably influencing this outcome. Oxidative stress, characterized by increased prooxidant levels and impaired antioxidant defense, was also prominent in adolescents with obesity. These novel findings underscore the complex interplay between obesity, lipid metabolism, redox status, and cellular aging, emphasizing the need for comprehensive approaches to mitigate the adverse health effects of obesity. However, further research is needed to fully understand the mechanisms underlying these associations and their implications for metabolic and cardiovascular diseases.

## Figures and Tables

**Table 1:** Socio-demographic, anthropometric, redox and lipid parameters, and plasma FAs of adolescents living with obesity or with healthy weight (control).

Parameter	Obese (*n* = 91)	Control (*n* = 44)	*p*
Sex (male/female)	45/46	22/22	ns
Age (year)	15 (11.5–16.0)	15 (12.5–16.5)	ns
BMI (kg/m^2^) ^aaa,bbb^	33.3 (27.3–37.0)	17.2 (16.1–21.1)	<0.001
Total cholesterol (mmol/L)	4.51 (3.78–5.08)	4.12 (3.38–4.72)	ns
LDL-cholesterol (mmol/L) ^aa^	2.62 (2.33–3.12)	2.14 (1.85–2.68)	0.045
HDL-cholesterol (mmol/L) ^aa^	1.30 (1.05–1.56)	1.58 (1.34–1.75)	0.028
Triglycerides (mmol/L) ^aa^	0.91 (0.51–1.20)	0.75 (0.63–1.00)	0.028
RFCVD ^aa^	3.4 (2.6–4.2)	2.4 (2.2–3.1)	<0.001
IA ^aa^	2.1 (1.5–2.7)	1.4 (1.0–1.9)	<0.001
TAS (mmol/L) ^aaa,bbb^	729(651–783)	439 (289–401)	<0.001
SOD (U/L) ^aaa,bbb^	95 (82–118)	139 (135–144)	<0.001
SHG (mmol/L) ^aaa,bbb^	0.352 (0.240–0.402)	0.455 (0.381–0.565)	<0.001
TOS (mmol/L) ^aaa,bbb^	100 (83–104)	61 (58–76)	<0.001
AOPPs (μmol/L) ^aaa,bbb^	78.1 (64.8–95.6)	44.9 (40.7–50.3)	<0.001
PAB (HK) ^aaa,bbb^	100.5 (94.7–110.5)	59.2 (51.0–67.7)	<0.001
PON (U/L) ^aaa,bbb^	171 (123–313)	261 (186–575)	<0.001
rLTL^aaa,bbb^	0.643 (0.440–0.871)	1.597 (1.520–1.819)	<0.001
14:0 (%) ^aaa,bbb^	2.8 (1.40–4.05)	0.70 (0.59–0.84)	<0.001
16:0 (%)	27.4 (26.06–28.28)	26.4 (23.69–27.45)	ns
18:0 (%) ^a,b^	11.49 (10.03–12.42)	12.35 (11.49–13.20)	0.01
18:1n-9 (%)	11.38 (10.13–12.23	11.61 (11.00–13.95)	0.045
18:2n-6 (%)	24.41 (22.61–26.07)	24.15 (21.85–27.24)	ns
20:3n-3 (%) ^a^	2.00 (1.90–2.23)	1.75 (1.65–2.05)	0.01
20:4n-6 (%) ^a,b^	10.21 (8.98–10.84)	10.98 (10.28–11.65)	0.045
22:1n-9 (%) ^aaa,bbb^	1.85 (1.46–3.47)	0.70 (0.45–0.95)	<0.001
22:4n-6 (%) ^a,b^	0.37 (0.28–0.70)	0.69 (0.41–0.90)	0.05
20:5n-3 (%) ^a,b^	0.88 (0.78–0.99)	1.15 (0.89–1.30)	0.002
22:5n-3 (%) ^a,b^	0.40 (0.31–0.51)	0.84 (0.60–0.92)	<0.001
22:6n-3 (%)	2.17 (1.77–3.03)	2.48 (2.05–2.58)	ns
Total n-3 PUFAs ^aa,bb^	5.45 (4.95–6.39)	6.22 (5.26–7.15)	0.003
Total n-6 PUFAs	34.99 (34.04–37.95)	35.82 (33.0–38.60)	ns
n-6-to-n-3 PUFA ratio ^aa,bb^	6.41 (5.60–7.15)	5.60 (4.91–6.40)	0.01

Data are median and interquartile range and were analyzed using the Mann–Whitney U test. ^a^ Differences between obese and control boys. ^b^ Differences between obese and control girls. ^a,b^ *p* < 0.05; ^aa,bb^ *p* < 0.01; ^aaa,bbb^ *p* < 0.001. Abbreviations: HDL, high-density lipoprotein; LDL, low-density lipoprotein; RFCVD, risk factor for cardiovascular disease; IA, index of atherosclerosis; ns, non-significant; TAS, total antioxidant status; SOD, superoxide dismutase: SHG, total sulfhydryl group; TOS, total oxidative status; AOPPs, advanced oxidation protein products; PAB, prooxidant–antioxidant balance; PON, paraoxanase; 14:0, myristic acid; 16:0, palmitic acid; 18:0, stearic acid; 18:1n-9, oleic acid; 18:2n-6, linoleic acid; 20:3n-3, eicosatrienoic acid; 20:5n-3, eicosapentaenoic acid (EPA); 20:4n-6, arachidonic acid; 22:1n-9, erucic acid; 22:4n-6, docosatetraenoic acid; 22:5n-3, docosapentaenoic acid (DPA); 22:6n-3, docosahexaenoic acid (DHA); n-3, omega-3; n-6, omega-6; PUFA, polyunsaturated fatty acid.

**Table 2:** Spearman’s correlations of FAs with lipid status and redox status parameters.

Parameter	14:0	16:0	18:0	18:1n-9	18:2n-6	20:4n-6	22:1n-9	22:4n-6	Total n-3	Total n-6	n-6/n-3
	Obese group										
Total cholesterol	-	0.250 *	-	-	-	0.218 *	−0.212 *	−0.231 *	-	-	-
LDL-cholesterol	-	0.264 **	-	-	-	-	-	-	-	-	-
HDL-cholesterol	-	-	-	-	-	-	−0.357 ***	-0.242 *	-	-	-
Triglycerides	0.266 **	-	-	-	-	-	-	-	-	-	0.243 *
RFCVD	0.250 *	-	-	-	-	-	-	-	-	-	-
IA	0.225 *	-	-	-	-	-	-	0.205 *	-	-	-
TOS	0.391 ***	-	-	-	0.225 *	-	-	-	-	0.290 **	-
AOPPs	0.470 ***	-	-	-	0.200 *	-	-	-	-	0.240 *	-
TAS	−0.331 *	-	-	-	-	-	-	-	-	-	-
SOD		-	-	-	-	-	-	-	-	-	-
SHG	0.385 ***	-	-	-	-	-	-	-	-	-	-
	Control group										
HDL- cholesterol	-	-	−0.410 **	-	-	-	-	-		-	-
TOS	-	-	0.349 **	-	-	0.526 *	-	-	−0.477 **	-	0.505 **
AOPPs	-	-	-	0.474 *	-	-	-	-	-	−0.608 **	-
TAS	-	-	-	−0.504 *	0.596 **	-	-	-	-	0.544 *	-
SOD	-	-	−0.264 *	-	-	-	-	-	0.527 **	-	−0.444 *
SHG	-	−0.295 *	-	-	−0.601 **	-	-		0.493 *	-	−0.517 *
PAB	-	-	-	-	-	-	-	-	−0.561 **	-	0.517 *
IMA	-	−0.341 **	-	-	-	-	-	-	−0.485 *	-	0.441 *

Values are Spearman correlation coefficients; * *p* < 0.05; ** *p* < 0.01; *** *p* < 0.001.

**Table 3:** Parameters that differed according to ratio of n-6 to n-3 PUFAs in adolescents with obesity.

Parameter	Low n-6/n-3 PUFA Ratio	High n-6/n-3 PUFA Ratio	*p*
rLTL	1.09 (0.79–1.28)	0.61 (0.41–0.89)	0.005
18:0	13.04 (10.91–13.63)	11.59 (10.17–12.34)	0.009
18:2n-6	20.54 (19.35–22.44)	24.51 (23.20–26.23)	<0.001
20:5n-3	0.78 (0.64–1.03)	0.50 (0.46–0.57)	0.028
n-3 PUFAs	7.44 (7.10–7.86)	6.33 (6.09–6.48)	<0.001
n-6 PUFAs	33.39 (32.25–34.88)	34.00 (32.59–35.90)	0.047
n-6-to-n-3 PUFA ratio	4.48 (4.17–4.75)	5.37 (5.13–5.62)	<0.001

Data were analyzed by the Mann–Whitney U test; low index, ratio lower then 5:1; high index, ratio above 5:1.

## Data Availability

Data is unavailable due to ethical restrictions.

## Supplementary Materials

The following supporting information can be downloaded at: https://www.mdpi.com/article/10.3390/biomedicines12040883/s1, Table S1A: Socio-demographic, anthropometric, redox and lipid parameters, and plasma FAs of adolescent boys living with obesity or with healthy weight (control); Table S1B: Socio-demographic, anthropometric, redox and lipid parameters, and plasma FAs of adolescent girls living with obesity or with healthy weight (control).
