# Supplementary material for: Circulating Fatty Acids Associate with Metabolic Changes in Adolescents Living with Obesity
Source: Biomedicines. 2024 Apr 17;12(4):883. doi: 10.3390/biomedicines12040883 (PMC11048623; doi:10.3390/biomedicines12040883)
Supplement: Supplementary file 1 [file biomedicines-12-00883-s001.zip › biomedicines-2908755-supplementary.pdf]

Table S1A. Socio-demographic, anthropometric, redox and lipid parameters and plasma FAs of adolescent boys living with obesity or with healthy weight (control).

Parameter	Obese boys	Control boys	p
Sex (male/female)	45	22	/
Age (yr)	14.0 (11.5 - 15.0)	14.5 (12.0-15.5)	ns
BMI (kg/m^2)	33.2 (29.0 -37.0)	18.6 (16.0 - 19.0)	< 0.001
Total cholesterol (mmol/L)	4.40 (3.70 - 4.80)	4.0 (3.57 -4.78)	ns
LDL-cholesterol (mmol/L)	2.48 (2.39 - 3.03)	1.87 (1.60-2.77)	0.01
HDL-cholesterol (mmol/L)	1.19 (0.97 - 1.44)	1.63 (1.45-1.72)	0.01
Triglycerides (mmol/L)	1.17 (0.75 - 1.30)	0.70 (0.59 - 0.79)	0.01
RFCVD	3.9 (2.9 - 4.2)	2.5 (2.1 - 2.9)	0.01
IA	2.4 (1.7 - 2.6)	1.2 (1.0 - 1.7)	0.01
TAS (mmol/L)	721(647 - 780)	437 (282 - 398)	< 0.001
SOD (U/L)	92 (80 - 115)	135 (132 - 140)	< 0.001
SHG (mmol/L)	0.349 (0.237 - 0.398)	0.450 (0.380 - 0.561)	< 0.001
TOS (mmol/L)	102 (85 - 105)	63 (60 - 78)	< 0.001
AOPPs (μmol/L)	78.6 (65.0 - 95.9)	46.9 (41.7 - 52.1)	< 0.001
PAB (HK)	100.9 (95.2 - 111.0)	59.8 (51.3 - 68.0)	< 0.001
PON (U/L)	174 (130 - 319)	266 (185 - 574)	< 0.001
rLTL	0.640 (0.435 - 0.869)	1.591 (1.525 - 1.814)	< 0.001
14:0 (%)	2.87 (1.45 - 4.09)	0.72 (0.61 - 0.89)	< 0.001
16:0 (%)	27.9 (26.11 - 28.30)	26.4 (23.71 - 27.48)	ns
18:0 (%)	11.54 (10.05 - 12.44)	12.39 (11.52 - 13.23)	0.023
18:1n-9 (%)	11.36 (10.20 - 12.26)	11.62 (11.04 - 13.99)	ns
18:2n-6 (%)	24.39 (23.61 - 26.04)	24.11 (21.84 - 26.21)	ns
20:3n-3 (%)	2.03 (1.92 - 2.25)	1.77 (1.69 - 2.07)	0.040

20:4n-6 (%)	10.23 (9.00 - 10.86)	11.0 (10.30 - 11.69)	0.040
22:1n-9 (%)	1.87 (1.48 - 3.50)	0.71 (0.47 - 0.98)	< 0.001
22:4n-6 (%)	0.40 (0.31 - 0.72)	0.73 (0.43 - 0.92)	0.05
20:5n-3 (%)	0.87 (0.74 - 0.97)	1.12 (0.87 - 1.28)	0.012
22:5n-3 (%)	0.38 (0.30 - 0.50)	0.81 (0.59 - 0.90)	0.015
22:6n-3 (%)	2.15 (1.74 - 3.00)	2.43 (2.04 - 2.56)	ns
Total n-3 PUFAs	5.43 (4.70 - 6.72)	6.13 (5.19 -6.81)	0.001
Total n-6 PUFAs	35.02 (32.92 - 37.62)	35.84 (32.87 - 38.82)	ns
n-6 to n-3 PUFA ratio	6.44 (5.60 - 7.15)	5.84 (4.91 - 6.40)	0.01

Abbreviations: HDL, high-density lipoprotein; LDL, low-density lipoprotein; RFCVD, risk factor for cardiovascular disease; IA, index of atherosclerosis; ns, non-significant; TAS, total antioxidant status; SOD, superoxide dismutase: SHG, total sulfhydryl group; TOS, total oxidative status; AOPPs, advanced oxidation protein products; PAB, prooxidant-antioxidant balance; PON, paraoxanase; 14:0, myristic acid; 16:0, palmitic acid; 18:0, stearic acid; 18:1n-9, oleic acid; 18:2n-6, linoleic acid; 20:3n-3, eicosatrienoic acid; 20:5n-3, eicosapentaenoic acid (EPA); 20:4n-6, arachidonic acid; 22:1n-9, erucic acid; 22:4n-6, docosatetraenoic acid; 22:5n-3, docosapentaenoic acid (DPA); 22:6n-3, docosahexaenoic acid (DHA); n-3, omega-3; n-6, omega-6; PUFA, polyunsaturated fatty acid.

Table S1B. Socio-demographic, anthropometric, redox and lipid parameters and plasma FAs of adolescent girls living with obesity or with healthy weight (control).

Parameter	Obese girls	Control girls	p
Sex (male/female)	46	22	/
Age (yr)	15.5 (12.5 - 16.5)	15 (12.5-16.0)	ns
BMI (kg/m^2)	33.3 (29.0 -39.2)	17.9 (15.5 - 19.2)	< 0.001
Total cholesterol (mmol/L)	4.59 (3.80 - 5.12)	4.20 (3.97-4.80)	ns
LDL-cholesterol (mmol/L)	2.69 (2.36 - 3.17)	2.31 (1.96-2.75)	ns
HDL-cholesterol (mmol/L)	1.27 (1.05 - 1.57)	1.42 (1.24-1.60)	ns
Triglycerides (mmol/L)	0.99 (0.59 - 1.23)	0.70 (0.61 - 0.91)	ns
RFCVD	3.4 (2.9 - 4.0)	3.0 (2.5 - 3.5)	ns
IA	2.1 (1.5 - 2.7)	1.7 (1.4 - 2.2)	ns
TAS (mmol/L)	735 (660 - 792)	440 (295 - 404)	< 0.001
SOD (U/L)	97 (84 - 120)	141 (138 - 146)	< 0.001
SHG (mmol/L)	0.348 (0.235 - 0.399)	0.451 (0.771 - 0.560)	< 0.001
TOS (mmol/L)	99 (80 - 101)	60 (55 - 74)	< 0.001
AOPPs (μmol/L)	77.7 (64.4 - 95.0)	43.7 (40.0 - 49.2)	< 0.001
PAB (HK)	100.0 (94.1 - 109.9)	58.7 (50.6 - 67.1)	< 0.001
PON (U/L)	168 (120 - 319)	258 (187 - 571)	< 0.001
rLTL	0.646 (0.449 - 0.878)	1.620 (1.527 - 1.825)	< 0.001
14:0 (%)	2.75 (1.38 - 4.00)	0.67 (0.52 - 0.80)	< 0.001
16:0 (%)	27.0 (25.96 - 28.21)	26.1 (23.60 - 26.98)	ns
18:0 (%)	11.39 (9.96 - 12.34)	12.30 (11.41 - 13.17)	0.02
18:1n-9 (%)	11.44 (10.20 - 12.29)	11.66 (11.012 - 14.901)	ns
18:2n-6 (%)	24.44 (22.99 - 26.14)	24.20 (21.91 - 26.29)	ns
20:3n-3 (%)	1.98 (1.87 - 2.10)	1.61 (1.64 - 2.06)	ns

20:4n-6 (%)	10.26 (9.02 - 10.89)	11.00 (10.32 - 11.70)	0.045
22:1n-9 (%)	1.87 (1.40 - 3.49)	0.69 (0.42 - 0.94)	< 0.001
22:4n-6 (%)	0.36 (0.25 - 0.68)	0.67 (0.40 - 0.90)	0.032
20:5n-3 (%)	0.80 (0.86 - 1.07)	1.19 (0.93 - 1.34)	0.04
22:5n-3 (%)	0.52 (0.32 - 0.65)	0.77 (0.62 - 0.95)	0.045
22:6n-3 (%)	2.19 (1.80 - 3.05)	2.49 (2.12 - 2.62)	ns
Total n-3 PUFAs	5.49 (4.85 - 6.87)	6.26 (5.31 -6.97)	0.003
Total n-6 PUFAs	35.06 (32.96 - 37.75)	35.87 (32.63 - 38.89)	ns
n-6 to n-3 PUFA ratio	6.38 (5.51 - 7.09)	5.73 (4.95 - 6.44)	0.01

Abbreviations: HDL, high-density lipoprotein; LDL, low-density lipoprotein; RFCVD, risk factor for cardiovascular disease; IA, index of atherosclerosis; ns, non-significant; TAS, total antioxidant status; SOD, superoxide dismutase: SHG, total sulfhydryl group; TOS, total oxidative status; AOPPs, advanced oxidation protein products; PAB, prooxidant-antioxidant balance; PON, paraoxanase; 14:0, myristic acid; 16:0, palmitic acid; 18:0, stearic acid; 18:1n-9, oleic acid; 18:2n-6, linoleic acid; 20:3n-3, eicosatrienoic acid; 20:5n-3, eicosapentaenoic acid (EPA); 20:4n-6, arachidonic acid; 22:1n-9, erucic acid; 22:4n-6, docosatetraenoic acid; 22:5n-3, docosapentaenoic acid (DPA); 22:6n-3, docosahexaenoic acid (DHA); n-3, omega-3; n-6, omega-6; PUFA, polyunsaturated fatty acid.
